# *Amigos de Fibro* (Fibro Friends): validation of an e-book to promote health in fibromyalgia

**DOI:** 10.1017/S1463423623000270

**Published:** 2023-05-31

**Authors:** Mateus Dias Antunes, Ana Carolina Basso Schmitt, Amélia Pasqual Marques

**Affiliations:** 1 Doctoral Student in the Program in Rehabilitation Sciences, Department of Physiotherapy, Speech-Language Pathology and Audiology, and Occupational Therapy, Faculty of Medicine, University of São Paulo, São Paulo, SP, Brazil; 2 Researcher, Department of Physiotherapy, Speech-Language Pathology and Audiology, and Occupational Therapy, Faculty of Medicine, University of São Paulo, São Paulo, SP, Brazil; 3 Researcher, Department of Physiotherapy, Speech-Language Pathology and Audiology, and Occupational Therapy, Faculty of Medicine, University of São Paulo, São Paulo, SP, Brazil

**Keywords:** fibromyalgia, educational technology, health education, health promotion, validation study

## Abstract

**Background:**

Educational strategies are necessary for the care of patients with fibromyalgia. The objective was to develop and validate an e-book to promote the health of individuals with fibromyalgia.

**Methods:**

Methodological research in which, initially, through a bibliographic survey, the available publications on the subject were analyzed. Then, this knowledge was used to build the theoretical content addressed, and the art and layout of the e-book were elaborated. In the third phase, validation of the constructed material, content specialists (*n* = 23), technicians (*n* =  23) and design specialists (*n* = 23), and individuals with fibromyalgia (*n* = 45) evaluated the e-book through the Delphi technique. For data collection, different questionnaires were used, according to the evaluation focus of each participant group, analyzed for reliability using Cronbach’s Alpha (αC) and agreement using the Content Validity Index (CVI).

**Results:**

In the global assessment of agreement from all groups of judges, the CVI presented a considerable minimum: content (0.79), technical (0.89), design (0.92), and target audience (0.97). Regarding reliability, all groups also had a αC within the acceptable range: content (0.960), technical (0.963), design (0.977), and target audience (1.08).

**Conclusions:**

The e-book was developed and validated in terms of content and relevance and can be used to promote the health of individuals with fibromyalgia.

## Introduction

Fibromyalgia is a chronic condition of unknown cause characterized by generalized bodily pain, fatigue, sleep disturbances, impaired cognition and anxiety (Wolfe *et al.*, 2010; Mascarenhas *et al.*, 2021). Its prevalence ranges from 0.2% to 6.6% in the general population (Marques *et al.*, 2017), and the condition causes disability with high direct costs (e.g. drug therapy and health care costs) and indirect costs (e.g. lost productivity) (Lacasse *et al.*, 2016). Diagnostic criteria for fibromyalgia have changed in recent years (Wolfe *et al.*, 2011).

There are several therapies available for fibromyalgia (Mascarenhas *et al.*, 2021). In this regard, the fibromyalgia treatment guidelines of the European League Against Rheumatism (Macfarlane *et al.*, 2017) recommend that non-pharmacological therapies based on exercise and patient education be tried first after the diagnosis of fibromyalgia. Although drug treatments appear to be effective, the evidence supporting the use of this type of treatment is relatively weak (Antunes and Marques, 2022a; Yin *et al.*, 2022).

Health education is one of the main items to enable the promotion of health in Primary Health Care in Brazil. It should prepare people to take control and responsibility for their own health and the health of their territory, as well as prepare them for empowerment, decision-making, participation, social control, and acting on the conditioning and determining agents of their health and quality of life (García-Ríos *et al.*, 2019; Antunes *et al.*, 2021b). The development of materials for health education is shown to be an efficient strategy for patients. Such an instrument encourages and stimulates the promotion of self-care. The e-book is a type of educational material, considered practical, of quick access, and that has digital resources that allow, for example, to increase the font size, make annotations, underline the text, and go to secondary sources with just a touch (Reberte *et al.*, 2012; Guaraná *et al.*, 2020).

Initially, for the elaboration of an e-book for health education, it is necessary to identify the real educational needs, since the material content of the e-book will be directly aligned with these needs, while the vocabulary must be consistent with the central message of this instrument. Good material is expected to have a planning of the central idea, with a correct, easy, understandable, and motivating message for the reader (Reberte *et al.*, 2012; Guaraná *et al.*, 2020). Thus, the validation of the e-book becomes a fundamental requirement to determine whether this instrument is suitable for distribution among individuals with fibromyalgia within the scope of primary health care. Therefore, the objective of this study was to develop and validate an e-book to promote the health of individuals with fibromyalgia.

## Methods

This is a methodological research (Polit and Beck, 2006). This type of study aims to elaborate, evaluate, and validate the technologies developed, in order to ensure their reliability for use in educational and care environments (Lobiondo-Wood and Haber, 2011). The guidelines for the construction and validation of guidance materials for health care proposed by Echer (Echer, 2005) were used.

In the first phase, through a literature review, the main publications available on multidisciplinary health promotion strategies for individuals with fibromyalgia were analyzed. The material intends to encourage the promotion of health and self-care in individuals with fibromyalgia, as well as helping them understand all aspects related to the syndrome. In its elaboration, it is essential to identify the reality of individuals, so that it has one of their needs and not just compliance with therapeutic requirements (Gozzo *et al.*, 2012).

So, in this part of the study, the synthesis of knowledge acquired during the literature review was used to build the theoretical knowledge to be addressed, as well as the discourse of health professionals and individuals with fibromyalgia collected in previous studies by the research group. Only then, with the help of a graphic designer, the art was created, through the making of figures and formatting, confirmation, and layout of the pages. In the preparation of the e-book, the aspects of language, illustration, and layout that the health professional should consider in the elaboration of educational materials, as proposed by Moreira, Nóbrega, and Silva (Moreira *et al.*, 2003) were taken into account in order to make them readable, understandable, effective and culturally relevant.

As the validity and reliability of the instruments are demonstrated, their quality is attested, the possibility of random errors is removed, and the credibility of their usability in practice is increased (Echer, 2005; Polit and Beck, 2006; Lobiondo-Wood and Haber, 2011). Then, the *Delphi* technique was used, which uses a questionnaire that can be repeated over and over until a convergence of the judges’ answers is reached. A consensus, that is, which represents the consolidation of the group’s intuitive judgment (Polit and Beck, 2006).

Regarding consultation with specialists in the area of interest, a sample calculation was performed to determine the number of judges obtained through the formula *n* = Za^2^.P(1-P)/e^2^. The stipulated values were Za (confidence level) = 95%, P (ratio of agreement of the judges) = 85%, and (accepted difference from what is expected) = 15% (Lopes MVO *et al.*, 2012; Galindo Neto *et al.*, 2017), which resulted in 22 judges. However, Vianna (Vianna, 1982) emphasizes the importance of having an odd number of specialists, in order to avoid a tie in opinions. After a systematic search described below, the sample of judges consisted of 23 participants by category group. We considered it coherent to divide the judges into three distinct categories: content judges (researchers/professors in the area of rheumatology, education and health promotion and educational technologies and/or validation of instruments); technical judges (professionals with experience in rheumatology, education, and health promotion); and judges with professional experience in design (Melo *et al.*, 2011).

The choice of judges in the area of interest was initially carried out by consulting the *Lattes* Platform of the National Council for Scientific and Technological Development (CNPq) (www.buscatextual.cnpq.br). The selection took place as follows: after accessing the website “*Plataforma Lattes*”, in the “Curriculum *Lattes*” window, the option “Search curriculum” was selected. In the advanced search tab, by subject, the following keywords were used: “Fibromyalgia” and “Health Promotion”. Preferably, doctors were chosen, rather than other researchers, with the understanding that professionals with more years of study and experience have a higher level of excellence. In addition, snowball sampling was also used (Costa, 2018). This technique is based on convenience sampling, widely used when the population is composed of individuals with characteristics that are difficult to find. In this case, an individual who meets the study participation criteria will be asked to nominate other participants (Polit and Beck, 2006).

In order to ensure an adequate assessment of the submitted content, it is essential that the judges be experts in the area of interest, which means that they must adopt a posture of valuing both their professional experience and the scientific knowledge acquired (Fehring, 1994; Jasper, 1994; Joventino, 2010). That being the case, and seeking to establish parameters for the selection of participants in this part of the study, a system for the classification of judges adapted from Joventino (Joventino, 2010) proposal was adopted, with the selection of those who reach a minimum score of five points.

The choice of technical judges was also carried out by snowball sampling (Costa, 2018). In the same way, these judges were chosen according to the adaptation made in the proposal by Joventino (Joventino, 2010). Lastly, design professionals with a degree in graphic technology design with at least 1 year of experience were invited. The selection of this group was also carried out under the snowball sampling criteria (Costa, 2018).

After the considerations of the three groups of experts, the necessary adjustments were made to the material, in order to proceed with the validation of the e-book by the target audience. This moment is special, because it was possible to identify what was not understood, what needs to be added, or even improved, in order to reduce the distance between what will be exposed and what will be learned (Echer, 2005). It is recommended that the portion of the target population that evaluates the e-book be 30 to 40 individuals (Beaton *et al.*, 2007). In that study, 45 individuals with fibromyalgia (importance of the odd number) with a medical diagnosis of fibromyalgia were evaluated through e-mail.

The inclusion criteria considered were: individuals of both sexes, aged 18 years or over and with a medical diagnosis of fibromyalgia, confirmed by the International Statistical Classification of Diseases and Related Health Problems (ICD-10) (DataSus, 2020), with code M79.7, and confirmed by an evaluator according to the ACR Classification Criteria, revised 2016 version (Wolfe *et al.*, 2016). Exclusion criteria were individuals undergoing physical therapy treatment or treated in the last three months; diagnosis of other conditions causing chronic pain (neuropathies, rheumatoid arthritis, osteoarthritis, spinal stenosis, or neoplasia); medically proven severe mental disorders (schizophrenia, psychosis, bipolar affective disorder, severe depression); visual or hearing impairment. All these criteria were considered self-reported by the participants. Individuals with fibromyalgia were invited to participate in the study through the Brazilian Association of Fibromyalgia Patients (ABRAFIBRO).

Considering that each group of participants had a specific focus on their evaluation, three instruments were used: the first, aimed at content and technical judges; the second, aimed at design judges, and the third, aimed at the target audience.

The Electronic Questionnaire for Content Assessment and Electronic Questionnaire for Technical Assessment sent to the content and technical judges was the Suitability Assessment of Materials (SAM), specific and widely used to assess the difficulty and convenience of educational materials. This instrument uses the standard Likert scale (0 = inadequate, 1 = partially adequate, 2 = adequate), in which there is a checklist for checking attributes related to content, writing style, graphic illustration, presentation, motivation, and cultural adequacy. For the e-book to be considered adequate, the result of the percentage calculation of scores obtained should be equal to or greater than 60% (Doak *et al.*, 1996; Moura *et al.*, 2017).

The Electronic Design Assessment Questionnaire aimed at design judges was built from the adaptation proposed by Souza (Souza, 2015). The first part of the instrument presents the evaluator’s identification; later, questions about the characteristics of the illustrations and, finally, a field for observations. In the valuation of the answers, the standard Likert scale was used, defined as follows: 1 = totally disagree, 2 = disagree, 3 = agree, 4 = totally agree. In this instrument, for validation of the instrument, a value greater than 0.78 is desirable (Borges *et al.*, 2013; Moura *et al.*, 2017).

Finally, the Electronic Assessment Questionnaire for the Target Audience for individuals with fibromyalgia was applied, built from Galdino (Galdino *et al.*, 2019), which presents subdivision items. The first part is composed of information on socio-economic profile; in the second part, it presents items regarding the domains of organization, writing style, appearance, and motivation, where a minimum agreement of 75% is required for positive responses and, in the third part, an open space is available for participants to express their personal opinions (Bispo *et al.*, 2012; Moura *et al.*, 2017).

After surveying all the suggestions made by specialists and individuals with fibromyalgia, the e-book was adapted in order to meet the needs and expectations of the population. Subsequently, the material was sent for Portuguese language proofreading (Echer, 2005).

Regarding the interpretation and analysis of the data, the professional information about the judges, as well as the socio-demographic and clinical data of the individuals with fibromyalgia, were organized in the *Excel* software 8.0, and the descriptive analysis was performed. As for the content validation of the e-book by the content and technical judges, the percentage of scores obtained was calculated through the total sum of the marked values divided by the total scores contained in the instrument. For the e-book to be considered adequate, the calculation result must be equal to or greater than 60% (Doak *et al.*, 1996).

In the validation of the e-book by expert design judges, the Content Validity Index (CVI) recommended by Waltz and Bausell (Waltz and Bausell, 1981) was used, which has been widely applied in this type of study. This method measures the degree of agreement of the experts in relation to aspects of the material; to do so, you must divide the number of answers marked with the values “3” and “4” (strongly agree and agree) by the total number of questions (Alexandre and Coluci, 2011). According to Lynn’s (Lynn, 1986) recommendations, the cutoff point used for the CVI was 0.78, both for each item answered and for the questionnaire as a whole. The analysis of the questionnaire applied to the target audience (individuals with fibromyalgia) was carried out in accordance with the recommendations of Teles (Teles, 2011), where it is essential that there be a minimum level of agreement of 75% in positive responses.

For the evaluation to be considered of good quality, it is necessary to present two basic conditions: reliability (measurement of consistency between evaluators) and agreement (degree to which two or more evaluators provide the same classification) (Matos, 2014). Thus, the reliability of the instruments measured in scale was analyzed using Cronbach’s Alpha (αC), which provides a reasonable measure of reliability in a single test. In this way, repetitions or parallel applications of a test are not necessary to estimate its consistency. This test is presented on a scale ranging from 0 to 1; in this case, values above 0.8 are accepted (Field, 2009).

As for the agreement among judges, the CVI that measures the proportion of the judges in agreement on a certain aspect evaluated was used. This method uses the Likert scale with scores from one to four. The index is calculated through the sum of agreement of items marked as positive responses, divided by the total responses (Alexandre and Coluci, 2011). In this sense, the CVI was calculated for each of the items listed in the blocks and for the total set of instrument items (sum of all CVI calculated separately, divided by the number of items), according to the recommendations of Lynn (Lynn, 1986), using the cutoff point of 0.78 (78%) for both calculations. All these data were calculated using the statistical program *Statistical Package for the Social Sciences (SPSS)*, version 22.0, serial no. 10101151049.

After surveying all the suggestions made by specialists and individuals with fibromyalgia, the e-book was adapted in order to meet the needs and expectations of the population. Subsequently, the material was sent for Portuguese language proofreading (Faria *et al.*, 2022). After analyzing the data, it was found that no more than one round of agreement would be necessary for all groups as proposed by the *Delphi* methodology, since in the first round, all groups reached a CVI above 0.78 and a αC above 0.8 in the global sum of the instruments (Joventino, 2010), thus being within acceptable values. The study was approved by the Research Ethics Committee of the Faculty of Medicine of the University of São Paulo, obtaining approval under number 3.197.778.

## Results

The final version of the e-book is comprised of 90 pages with the title “Amigos de Fibro: Promovendo a Saúde na Fibromialgia”. The e-book was created using the Canva software, a graphic design platform that allows users to create different types of visual content. In addition, it features graphic illustrations taken from Envato Elements, a subscription platform for graphic design items.

The material was divided into 10 blocks with the following themes: getting to know fibromyalgia (chapter 1); health promotion and care (chapter 2); family and work (chapter 3); body practices and physical activities (chapter 4); healthy and adequate nutrition (chapter 5); health and well-being (chapter 6); pharmacological approach (chapter 7); integrative and complementary practices (chapter 8); occupational performance (chapter 9) and sleep quality (chapter 10).

The educational content refers to self-care measures that promote the health and quality of life of individuals with fibromyalgia. In order to enrich the content, improve readability and general reader captivation of the e-book, and offer the target audience the opportunity to delve deeper into the topic, references and links were added at the end of the material, which gives access to the entirety of articles, books, theses, and websites that also support the theoretical framework of this material. The final version of e-book is available at: http://www.amigosdefibro.com.br.

Regarding the content judges, the majority were female (56.5%), aged between 31 and 40 years (39.2%), married or living with a partner (53%), white race/color (82 .7%), with teaching experience (100%), graduated between 11 and 20 years (39.2%), with an academic doctorate as their highest degree (39.2%) and all (100%) were teaching and with practical experience in the area of interest between 1 and 10 years (47.8%). Regarding their professions, 17.4% were physiotherapists, 13% rheumatologists, and the other professions had 8.7% (nurse, psychologist, nutritionist, occupational therapist, speech therapist, pharmacist, naturologist, and social worker).

As for the technical judges, the majority were female (65.2%), aged between 20 and 40 years (69.8%), married or living with a partner (65.2%), white race/color (69 .6%), with specialization in the area of interest (82.6%), recent practice in the area of interest (87%), as well as 69.6% participated in a course or training in the area of interest in the last 5 years, presented work in the area of interest in scientific events in the last 5 years, as well as having teaching experience in the area of interest.

The majority of the design judges were male (65.2%), aged between 20 and 30 years (52.2%), single (60.9%), white race/color (69.6%), with training time of 1 to 5 years (87%) and worked as designers in a publishers and advertising agencies (78.2%). Finally, most of the target audience judges were female (91%), aged between 31 and 40 years (38%), married or living with a partner (65%), white race/color (53%), with incomplete higher education (49%), monthly income of 1 to 2 minimum wages (60%), consider their health poor (49%) and use three or more medications a day (67%).

Regarding the process of judging the items that make up the e-book by the content judges, none of them was evaluated as totally inadequate. As shown in Table 1, the majority (61.1%) obtained a considerable minimum agreement index by the CVI. However, in the global assessment by the content judges, the CVI of the e-book presented a considerable minimum (0.79) and also an acceptable αC (0.960).

**Table 1.** Content specialists’ assessment of content, language, graphic illustrations, presentation, stimulus/motivation, and cultural adequacy of the e-book

Items	Inadequate		Partially adequate		Adequate		CVI
	***n***	%	***n***	%	***n***	%	
**Contents**							
The objective is evident, facilitating immediate understanding of the material	–	–	5	21.7	18	78.3	0.78
The content addresses information related to behaviors that help promote health in fibromyalgia	–	–	–	–	23	100	10.0
The proposal of the material is limited to the objectives, so that the reader can reasonably understand in the time allowed.	–	–	8	34.8	15	65.2	0.65
**Language**							
Reading level is adequate for reader comprehension	–	–	5	21.7	18	78.3	0.78
The conversational style facilitates the understanding of the text	–	–	6	26.1	17	73.9	0.73
Information is conveyed within a clear context	–	–	5	21.7	18	78.3	0.78
The vocabulary uses common words	–	–	7	30.4	16	69.6	0.69
Learning is facilitated by topics	–	–	4	17.4	19	82.6	0.82
**Graphic illustrations**							
The cover attracts the reader’s attention and portrays the purpose of the material	–	–	2	8.7	21	91.3	0.91
The illustrations present key visual messages so that the reader can understand the main points on their own, without distractions.	–	–	6	26.1	17	73.9	0.73
The illustrations are relevant	–	–	7	30.4	16	69.6	0.69
**Presentation**							
The organization of the material is adequate	–	–	5	21.7	18	78.3	0.78
The font size and type promote a pleasant reading	–	–	6	26.1	17	73.9	0.73
**Stimulation/motivation**							
The text and/or figures interact with the reader, getting them to solve problems, make choices and/or demonstrate skills	–	–	6	26.1	17	73.9	0.73
The desired patterns of behavior are modeled or well demonstrated	–	–	5	21.7	18	78.3	0.78
There is motivation for self-efficacy. That is, people are motivated to learn because they believe that the tasks and behaviors are feasible.	–	–	–	–	23	100	10.0
**Cultural adequacy**							
The material is culturally appropriate in the logic, language and experience of the target audience	–	–	4	174	19	82.6	0.82
It presents culturally adequate images and examples	–	–	4	17.4	19	82.6	0.82

**Caption:** CVI = content validity index.

As for the process of judging the items that make up the e-book by the technical judges, none of them was evaluated as totally inadequate. As shown in Table 2, all items (100%) had a considerable minimum agreement rate by the CVI. In the global evaluation by the technical judges, the e-book obtained a CVI of 0.89 and also an acceptable αC (0.963).

**Table 2.** Evaluation of technical experts regarding the content, language, graphic illustrations, presentation, stimulus/motivation, and cultural adequacy of the e-book

Items	Inadequate		Partially adequate		Adequate		CVI
	***n***	%	***n***	%	***n***	%	
**Contents**							
The objective is evident, facilitating immediate understanding of the material	–	–	2	8.7	21	91.3	0.91
The content addresses information related to behaviors that help promote health in fibromyalgia	–	–	–	–	23	100	10.0
The proposal of the material is limited to the objectives, so that the reader can reasonably understand in the time allowed.	–	–	5	21.7	18	78.3	0.78
**Language**							
Reading level is adequate for reader comprehension	–	–	2	8.7	21	91.3	0.91
The conversational style facilitates the understanding of the text	–	–	3	13	20	87	0.86
Information is conveyed within a clear context	–	–	3	13	20	87	0.86
The vocabulary uses common words	–	–	4	17.4	19	82.6	0.82
Learning is facilitated by topics	–	–	1	4.3	22	95.7	0.95
**Graphic illustrations**							
The cover attracts the reader’s attention and portrays the purpose of the material	–	–	–	–	23	100	10.0
The illustrations present key visual messages so that the reader can understand the main points on their own, without distractions.	–	–	4	17.4	19	82.6	0.82
The illustrations are relevant	–	–	4	17.4	19	82.6	0.82
**Presentation**							
The organization of the material is adequate	–	–	2	8.7	21	91.3	0.91
The font size and type promote a pleasant reading	–	–	3	13	20	87	0.86
**Stimulation/motivation**							
The text and/or figures interact with the reader, getting them to solve problems, make choices and/or demonstrate skills	–	–	3	13	20	87	0.86
The desired patterns of behavior are modeled or well demonstrated	–	–	3	13	20	87	0.86
There is motivation for self-efficacy. That is, people are motivated to learn because they believe that tasks and behaviors are feasible.	–	–	1	4.3	22	95.7	0.95
**Cultural adequacy**							
The material is culturally appropriate in the logic, language and experience of the target audience	–	–	1	4.3	22	95.7	0.95
It presents culturally adequate images and examples	–	–	1	4.3	22	95.7	0.95

**Caption:** CVI = content validity index.

Regarding the process of judging the items that make up the e-book by the design judges, none of them were evaluated with “totally disagree”. As shown in Table 3, all (100%) had an agreement index greater than the cutoff score considered acceptable. In the global evaluation by the design judges, the e-book had a CVI of 0.92 and an acceptable αC (0.977).

**Table 3.** Evaluation of specialists in the area of design, regarding the characterization of the graphic illustrations of the e-book

Items	Judgment								
	Strongly disagree		Disagree		Agree		Strongly agree		CVI
	***n***	%	***n***	%	***n***	%	***n***	%	
Are appropriate for the target audience	–	–	1	4.3	13	46.5	9	39.1	0.95
They are clear and convey easy understanding.	–	–	1	4.3	11	47.8	11	47.8	0.95
They are in adequate quantity and sizes in the material.	–	–	2	8.7	9	39.1	12	52.2	0.91
They are related to the text of the album and elucidate the content.	–	–	2	8.7	5	21.7	16	69.6	0.91
The colors and shapes of the figures are suitable for the type of material.	–	–	1	4.3	15	65.2	7	30.4	0.95
They portray the daily life of the target audience.	–	–	2	8.7	7	14	14	60.9	0.91
The arrangement of the images is in harmony with the text.	–	–	2	8.7	12	14	9	39.1	0.91
The images help in exposing the theme and follow a logical sequence.	–	–	2	8.7	10	14	11	47.8	0.91
They contribute to changing the behavior and attitudes of the target audience	–	–	2	8.7	4	14	17	73.9	0.91
They are relevant to the understanding of the content by the target audience.	–	–	2	8.7	4	14	17	73.9	0.91

**Caption:** CVI = content validity index.

In the judgment process of the items that compose the e-book by the target audience judges, none of them was evaluated as totally inadequate. As shown in Table 4, of the total number of items evaluated, 46% had only positive responses, 31% had negative responses, and 23% had impartial responses. However, in the global evaluation by the target audience judges, the e-book obtained a CVI of 0.97 and an acceptable αC (1.08), within acceptable values.

**Table 4.** Assessment by the target audience, regarding the organization, writing style, appearance, and motivation of the booklet

Items	Positive responses		Negative responses		Impartial responses		CVI
	***n***	%	***n***	%	***n***	%	
**Organization**							
Did the cover catch your eye?	45	100	–	–	–	–	10.0
Is the content sequence adequate?	44	97.8	–	–	1	2.2	0.97
Is the structure of the educational booklet adequate?	44	97.8	–	–	1	2.2	0.97
**Writing style**							
Are the sentences (easy to understand/difficult to understand/don’t know)?	41	91.1	4	8.9	–	–	0.91
Is the written content (clear/confusing/don’t know)?	44	97.8	1	2.2	–	–	0.97
Is the text (interesting/uninteresting/don’t know)?	45	100	–	–	–	–	10.0
**Appearance**							
Are the illustrations (simple/complicated/don’t know)?	44	97.8	1	2.2	–	–	0.97
Do the illustrations serve to complement the text?	44	97.8	1	2.2	–	–	0.97
Do the pages or sections look organized?	45	100	–	–	–	–	10.0
**Motivation**							
In your opinion, will anyone with fibromyalgia who reads this educational material understand what it is about?	42	93.3	–	–	3	6.7	0.93
Did you feel motivated to read the educational material to the end?	45	100	–	–	–	–	10.0
Does the educational material address the issues necessary for adolescents to adopt a healthier lifestyle?	45	100	–	–	–	–	10.0
Did the educational material suggest that you act or think about preventing the worsening of fibromyalgia symptoms?	45	100	–	–	–	–	10.0

**Caption:** CVI = content validity index.

## Discussion

Through this study, an educational e-book was developed and validated in order to promote self-care to improve the health of individuals with fibromyalgia. Educational materials, when prepared by experts in the field and through scientific bases, contribute to a better understanding of conditions and their treatments, being attractive and useful to the intended audience (Faria *et al.*, 2022).

Communication in health is considered the employment of methods to inform, plan, influence, and even to execute decisions that usher health improvement, making it an essential tool in health promotion because it has the capacity to promote knowledge, bring solutions to health issues, as well as induce new perceptions and transform attitudes (Silva *et al.*, 2006; Guaraná *et al.*, 2020). This is what is sought in the treatment of fibromyalgia and is recommended according to international guidelines for its management (Macfarlane *et al.*, 2017).

Patient education is a key component in managing fibromyalgia, as with other chronic conditions. An open presentation and discussion between patients and care providers is essential. Involving the patient in reviewing care options and participating in the development of a plan with the greatest likelihood of being followed are also goals pursued in the management of fibromyalgia (Chokshi, 2022).

From this perspective, health professionals must be able to identify health needs and intervene efficiently to improve the quality of life of these individuals. In this sense, attention should be paid to changes in care paradigms, to interventions in the health promotion model, to the strategies of educational actions required in care, in which all participants in the action are invited to get involved at the starting point in the establishment of goals for self-care. So, care through health education is capable of promoting empowerment for individuals with fibromyalgia (Miranda *et al.*, 2016).

Empowerment is an educational process designed to help patients develop the knowledge, skills, attitudes, and self-knowledge necessary to effectively assume responsibility for decisions about their health (Feste and Anderson, 1995). Patients who are more informed, involved, and accountable (empowered) interact more effectively with health professionals trying to carry out actions that produce health results (Wagner, 1998; Taddeo *et al.*, 2012).

Most experts agree that an educational treatment component is helpful, if not necessary, in treating fibromyalgia. These educational programs aim to increase understanding of the complex nature of interactions between neurobiological processes, behaviors such as sleep or activity levels, and symptoms. These programs have varied focuses but generally attempt to lessen the stigma often associated with fibromyalgia and similar disorders (Hassett and Gevirtz, 2009; Gómez-de-Regil, 2021).

Providing educational materials that promote basic self-management techniques to perform daily activities and manage symptoms, as well as suggesting ways to incorporate wellness activities into daily life, should be one of the main forms of care in fibromyalgia. In addition, it is important that caregivers be aware of these educational materials and use them in their clinical practice (Arnold *et al.*, 2012; Arnold and Clauw, 2017).

Continuing education after diagnosis is a key goal in fibromyalgia. As part of this continuing education, the importance of having good quality educational materials should be highlighted so that they can be disseminated to all patients (Arnold *et al.*, 2012).

To the best of our knowledge, there are no studies on the development and validation of an e-book to promote the health of individuals with fibromyalgia. Thus, the addition of this e-book to fibromyalgia treatment protocols is necessary. Especially when combined with other modalities, such as physical exercise, sleep hygiene, and psychological support, it tends to increase the effectiveness of care (Hassett and Gevirtz, 2009).

This e-book was built from the opinions and experiences of patients with fibromyalgia, as well as health professionals. This differential in the development of the e-book corroborates the scientific literature, especially in the context of preparing educational materials, as this listening can improve the content of the material to promote self-management of care for patients with fibromyalgia, helping to improve adaptive behaviors and incorporate them into daily care practices (Montesó-Curto *et al.*, 2018).

Fibromyalgia treatment methods differ from patient to patient, as there is no standardization of treatment protocols. Methods that use a single treatment do not produce satisfactory clinical results (Saral *et al.*, 2016), so the *Amigos de Fibro* (*Fibro Friends*) e-book was developed to be used in support of the care already offered to these patients. The *Amigos de Fibro* (*Fibro Friends*) e-book does not seek to be the only treatment and should be used as a complement to care approaches. According to the scientific literature, no one treatment works for all symptoms of the syndrome, and fibromyalgia is usually treated using a combination of approaches, including medication, exercise, sleep hygiene, and psychological support. In addition, treatment requires patients to be active participants in promoting their care, so the e-book comes with the purpose of empowerment (Arnold *et al.*, 2012).

The use of new technologies is growing in health communication for health promotion, disease prevention, and health care delivery. In addition, technologies help self-manage a condition, provide social support, or provide help in making health decisions (Yuan *et al.*, 2021). Online educational strategies have been carried out. Contrary to popular belief, online educational intervention has been shown to be effective, especially in improving patient knowledge about the disease, health outcomes, and psychological variables. Some studies use specific websites to provide educational information to fibromyalgia patients (Conversano *et al.*, 2019). In addition, there are targeted interventions in *eHealth* (health care supported by information and communication technology solutions) that have been applied in Brazil. The first one in Brazil was the *ProFibro* application, which was developed and has shown positive results in improving quality of life, symptoms, and self-care in this population (Yuan and Marques, 2018).

Regarding non-pharmacological treatments for fibromyalgia, health education strategies have been successfully adopted in most cases (Pereira Pernambuco *et al.*, 2018; Antunes *et al.,* 2021b). They can induce healthy habits, coping strategies, and empowerment, and their benefits have been demonstrated in some studies (Díaz-Cerrillo *et al.*, 2016; Yuan *et al.*, 2021). However, they were always carried out through printed material, and the e-book format is not found in the scientific literature. In addition, the studies carried out have different characteristics regarding the direction of the education and the development of intervention sessions (García-Ríos *et al.*, 2019; Antunes *et al.*, 2021b).

It is important that health professionals be trained and prepared to apply educational interventions for individuals with fibromyalgia and disseminate this possibility of care throughout the health network. The *Amigos de Fibro* (*Fibro Friends)* e-book emerges as a good alternative to promote health in the context of primary health care in Brazil. Primary health care providers should also consider including educational technologies, especially mild ones, in the ongoing care of patients with fibromyalgia (Arnold *et al.*, 2012). Thus, works like this stand out as facilitators in the process of understanding the health-promoting attitudes of individuals with fibromyalgia, facilitating communication, and promoting better control of the syndrome’s symptoms (Faria *et al.*, 2022). *Amigos de Fibro (Fibro Friends)* began to be shown in national and international scientific literature, as well as in scientific events with the aim of disseminating scientific knowledge and the application of *Amigos de Fibro (Fibro Friends)* in different spaces and contexts. In addition, the great project Amigos de Fibro has already published its development (Antunes *et al.,* 2022c) and validation (Antunes *et al.,* 2022d) version in national and international journals, as well as presented at various events related to rheumatology and public health (Antunes *et al.,* 2021e; Antunes *et al.,* 2021f; Antunes *et al.,* 2022g; Antunes *et al.,* 2022h).

### Study limitations

Despite presenting several positive characteristics, excellent content, and many advantages, the e-book as a means of dissemination has peculiarities that bring certain limitations to the work. In Brazil, there are still locations without access to the internet. Therefore, access to technology is precarious or even non-existent, which reduces the reach of the material and the dissemination of educational content. Furthermore, the patient-therapist alliance is likely crucial to reconceptualizing pain and changing the patient’s beliefs around the illness. The e-book makes the exchange between patient and professional impossible. However, it must be added to interventions already performed by the patient.

## Conclusion

The e-book was designed and validated for its content and appearance by groups of judges and target audiences. The proposal to offer information on self-care in fibromyalgia presented practical and scientific relevance, having the potential to contribute to the empowerment and health promotion of these individuals. It is believed that the e-book is, in addition to an educational material, an instrument to improve communication and health empowerment for individuals with fibromyalgia.

### Practical implications

The use of the e-book by individuals with fibromyalgia can promote knowledge regarding care and practices to control the symptoms of the syndrome, consequently subsidizing support through a light educational technology that promotes the health and quality of life of these individuals.
